# Torsade de pointes associated with long-term antiretroviral drugs in a patient with HIV: a case report

**DOI:** 10.3389/fphar.2023.1268597

**Published:** 2023-10-31

**Authors:** Xuechun Mu, Yujiao Duan, Qiuhua Xu, Sa Wang, Guiju Gao, Ning Han, Hongxin Zhao

**Affiliations:** ^1^ Emergency Department of Infectious Diseases, Beijing Ditan Hospital, Capital Medical University, Beijing, China; ^2^ Department of Infectious Disease, Beijing Ditan Hospital, Capital Medical University, Beijing, China

**Keywords:** human immunodeficiency virus, drug interaction, QT prolongation, torsades de pointes, lopinavir, ritonavir, terfenadine

## Abstract

With the improving life expectancy of patients with human immunodeficiency virus (HIV), there is an increasing health concern of potential toxicity and drug interactions of long-term antiretroviral therapies. We describe a female patient with HIV, who was admitted to the emergency department following an unexplained loss of consciousness. This patient had been on antiretroviral therapy comprising tenofovir disoproxil fumarate, lamivudine, and lopinavir/ritonavir for 12 years. Coincidentally, she had been prescribed terfenadine for urticaria recently. After 3 days on this medication, she suddenly lost her consciousness, with a distinctive electrocardiogram alteration characterized by QT prolongation and torsade de pointes. This symptom recurred several times over a span of 2 days. We postulate that the primary instigator was an elevated concentration of terfenadine, which can be traced back to her antiretroviral therapy regimen comprising lopinavir/ritonavir. This drug is known to impede the metabolism of cytochrome P450 3A4 substrates and consequently elevate terfenadine concentrations.

## 1 Introduction

With the improving life expectancy of patients with human immunodeficiency virus (HIV), there is an increasing health concern of potential toxicity and drug interactions of long-term antiretroviral drugs (ARVs) (Margolis et al., 2014; Clutter et al., 2016). Clinicians are now more frequently navigating complex scenarios of drug-drug interactions stemming from concurrent diseases (Okeahialam et al., 2006; Palella et al., 2006). Although superior antiretroviral therapy (ART) regimens with fewer adverse effects have been recommended, many developing countries including China predominantly rely on the more accessible and cost-effective combination of two nucleoside reverse transcriptase inhibitors combined with either non-nucleoside reverse transcriptase inhibitors or protease inhibitors (PIs) as the first-line ART (Ghosn et al., 2018; Cao et al., 2020).

Lopinavir (LPV) is one of PIs, that can inhibit the HIV type 1 protease highly and selectively. Ritonavir robustly inhibits hepatic cytochrome P450 (CYP) 3A4 enzymes, enhancing blood concentration and effectiveness against HIV when co-formulated as lopinavir/ritonavir (LPV/r) (Cvetkovic and Goa, 2003; Vogel and Rockstroh, 2005). Terfenadine, an H1-antihistamine, has fallen out of favor due to its risk of dose-dependent cardiotoxicity (Li et al., 2022). In the presence of significant hepatic diseases or agents inhibiting CYP enzymes, the level of terfenadine would be elevated, potentially inducing long QT syndrome (LQTS) as well as torsade de pointes (TdP) (Li et al., 2017). However, the studies on TdP induced by drug interactions are few, with no reports on interactions between ARVs and terfenadine. Here, we describe a patient with HIV who experienced a fatal arrhythmia and a sudden loss of consciousness due to terfenadine toxicity, a consequence of its drug interaction with LPV/r.

## 2 Case presentation

**FIGURE 1:**
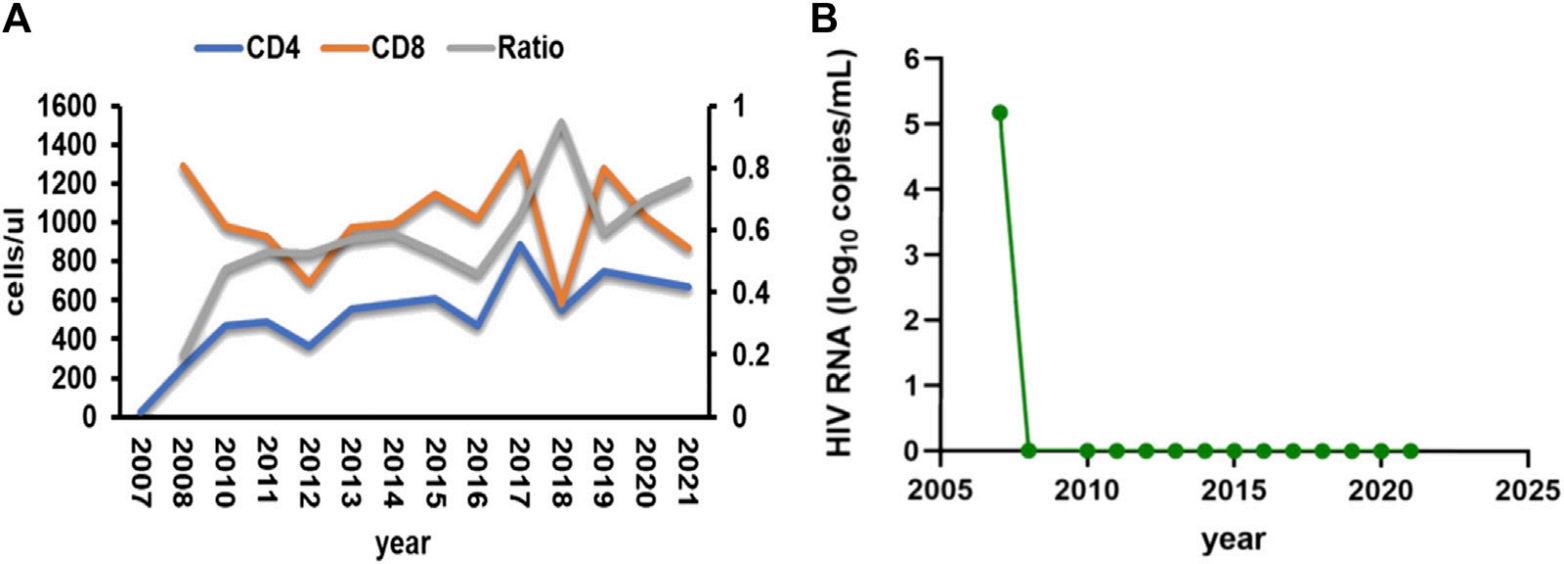
**(A)** Patient’s CD4 T cells, CD8 T cells and the ratio of them during the follow-up time. **(B)** Patient’s HIV RNA load during the follow-up time.

A female patient, aged 38, with a medical history of acquired immunodeficiency syndrome undergoing long-term ART in our hospital, experienced an unexpected loss of consciousness and limb convulsion during her sleep on 27 July 2021, as observed by her husband. Then she was admitted to the emergency department. On her way to the hospital, she suffered another episode of unconsciousness. At that time, the heart monitor in the ambulance captured ventricular fibrillation, which was resolved via defibrillation, restoring her consciousness. Since 2009, She took the ART of tenofovir disoproxil fumarate (TDF) (300 mg/d), lamivudine (3TC) (300 mg/d), and LPV/r (400/100 mg, twice a day) and maintained a stable immune status and undetectable HIV RNA (Figure 1). With no known cardiac history, her annual check-ups consistently showed normal electrocardiogram (ECG) and liver function. She denied recent travel history but had developed urticaria, presumably due to food allergies. Unaware of her medication history, an external dermatologist prescribed her terfenadine (60 mg, three times a day) and prednisone (30 mg/d) on 24 July 2021. Recurrent loss of consciousness happened on the third day after terfenadine therapy.

**FIGURE 2:**
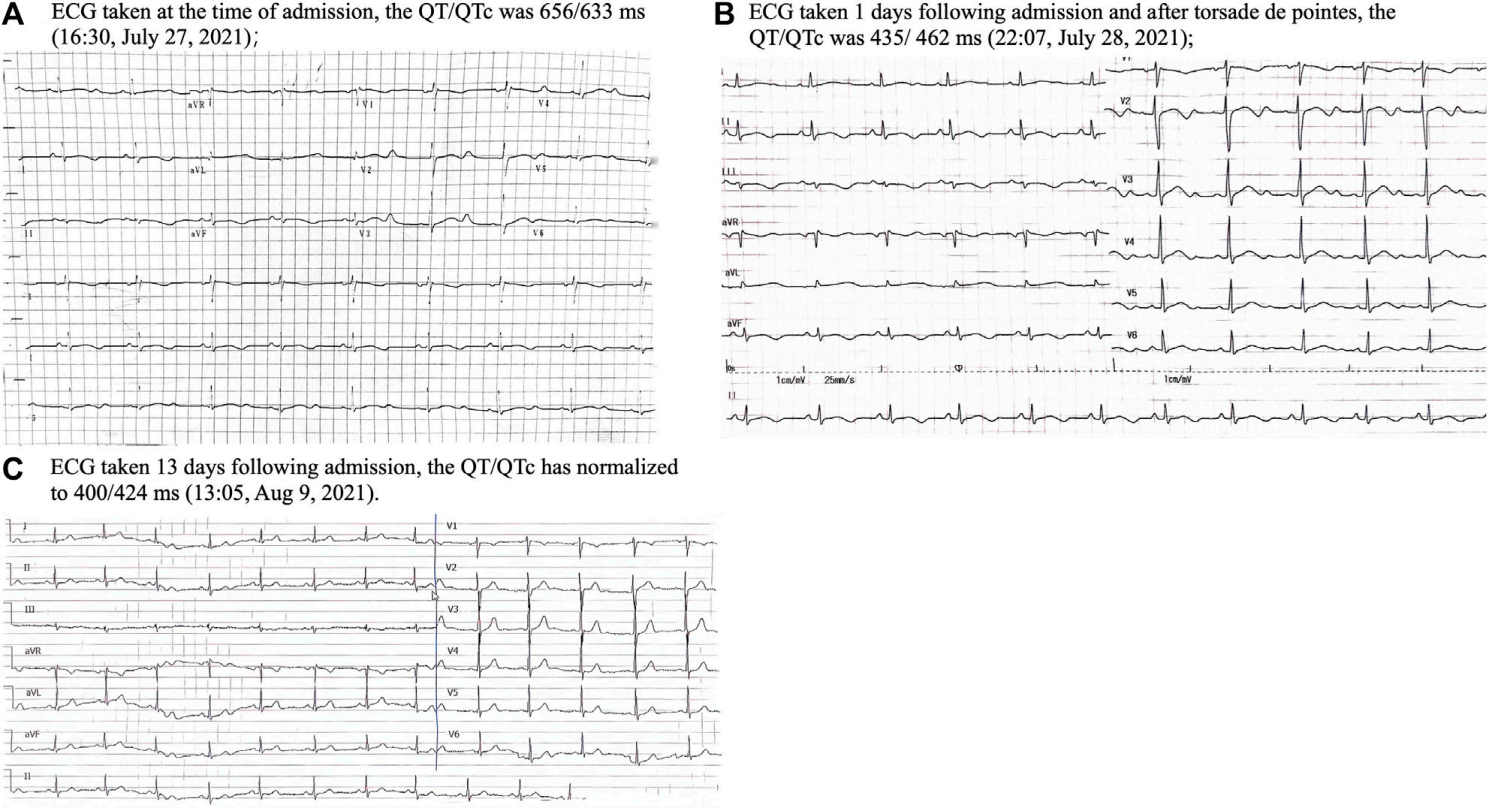
The change of twelve-lead ECG while the patient was hospitalized.

Upon admission, the vital signs on presentation showed a heart rate of 56 per minute, blood pressure of 118/67 mmHg, temperature of 36°C and respiratory rate of 18 per minute. A physical examination revealed a lethargic patient with no focal neurological deficits, clear lung sounds and a soft abdomen, but dark red rashes in her extremities. ECG revealed sinus bradycardia with a notably prolonged corrected QT (QTc) of 633 ms (Figure 2A). Laboratory tests showed hypoalbuminemia of 3.48 g/dL, and slight elevation of aspartate transaminase (63.80 U/L) as well as alanine transaminase (56.60 U/L). Her blood routine examination, serum electrolytes and renal function were within normal limits, although potassium (3.74 meq/L) and magnesium (0.80 meq/L) were near the lower threshold. Echocardiogram and cerebrospinal fluid tests showed normal. Non-contrast computerized tomography of the head and chest did not show any acute abnormality. Given the suspected drug-induced QT prolongation and severe arrhythmias, all medications, including terfenadine and ARVs, were withdrawn.

**FIGURE 3:**
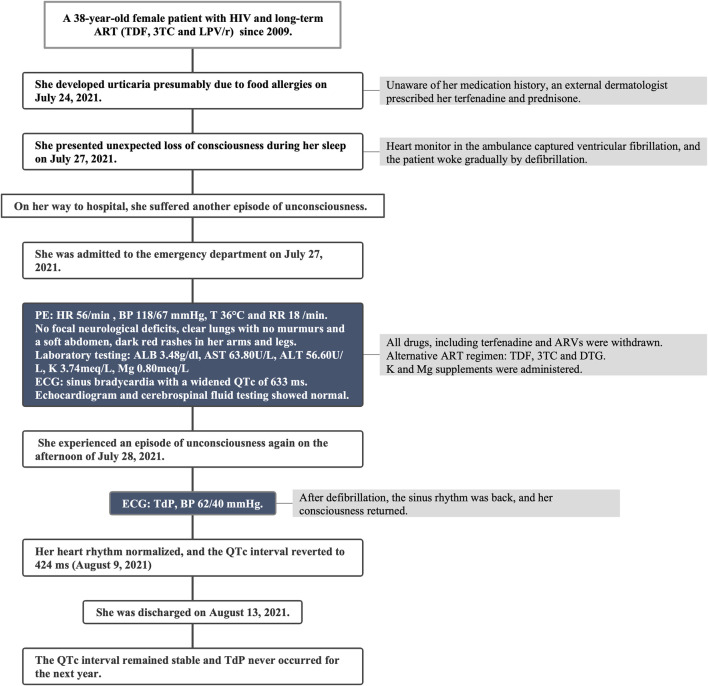
Timeline of the presented case report. HIV, human immunodeficiency virus; TDF, tenofovir disoproxil fumarate; 3TC, lamivudine; LPV/r, lopinavir/ritonavir; PE, physical examination; HR, heart rate; BP, blood pressure; T, temperature; RR, respiratory rate; ALB, albumin; AST, aspartate transaminase; ALT, alanine transaminase; K, potassium; Mg, magnesium; ECG, electrocardiogram; QTc, corrected QT; DTG, dolutegravir; TdP, Torsade de Pointes.

On the afternoon of 28 July 2021, the patient suffered another episode of unconsciousness. ECG monitoring during this episode exhibited a typical TdP with oscillation of the QRS axis around the baseline, and her blood pressure dropped to 62/40 mmHg. Following defibrillation, the sinus rhythm was back, and her consciousness returned. Regrettably, the TdP failed to be recorded on ECG. The subsequent 24-h continuous ECG monitoring captured no TdP or other malignant arrhythmias, although the QT prolongation persisted (Figure 2B). Potassium and magnesium supplements were administered. Neither anti-arrhythmic drugs nor atrial ventricular pacing were used. To mitigate the drug-interaction toxicity and sustain the prior ART efficacy, an alternative ART regimen was initiated, excluding CYP450 system inhibitors: TDF, 3TC and dolutegravir. After 1 week on 9 August 2021, her heart rhythm normalized, with the QTc interval reverting to 424 ms (Figure 2C). This patient was discharged on 13 August 2021. Over the subsequent year, her QTc interval remained stable and there was no recurrence of TdP. This streamlined treatment facilitated her adherence to long-term ART. Timeline of the presented case is shown in Figure 3.

## 3 Discussion

TdP, an infrequent polymorphic ventricular tachycardia, is characterized by a distinct ECG pattern that shows a progressive alteration in the magnitude and rotation of the QRS complexes around the isoelectric line. The risk for TdP is notably increased with QTc prolongation, especially, when the QTc exceeds 500 ms (Sauer et al., 2007). The most common cause of acquired LQTS, which can lead to TdP and sudden cardiac death is drugs (Roden, 2004). Cardiotoxic drugs that inhibit the rapid component of the delayed rectifier potassium current (I_Kr_) can prolong the action potential duration and elevate the risk of early afterdepolarizations. Blocking I_Kr_ results in an extended ventricular action potential duration, leading to an influx of sodium or a decreased efflux of potassium. This ionic imbalance extends the repolarization phase, resulting in a prolonged QT interval and TdP (Drew et al., 2010; Ali et al., 2021). Notably, apart from anti-arrhythmic drugs, such as quinidine (Reynolds et al., 1976), disopyramide (Aarskog et al., 1992), and procainamide (Vlasses et al., 1986), several non-cardiac drugs, such as antibiotic drugs (Oberg et al., 1995), probucol (Gohn et al., 1992), methadone (Deamer et al., 2001; Krantz et al., 2002), and anticancer drugs (Schwartz and Woosley, 2016) have been reported to induce TdP.

Terfenadine, a cardiotoxic antihistamine, was removed from the market in the United States and several Europe countries during the 1990s due to associated risks (Monahan et al., 1990). However, it remains in use in China and some developing countries to treat rhinallergosis and urticaria. QT prolongation induced by terfenadine is time and concentration dependent. Its recommended dose is below 60 mg twice a day. Overdosing can induce LQTS and TdP (Hondeghem et al., 2011). Terfenadine is catalyzed by CYP3A in the liver before excretion (Ling et al., 1995). There has been a documented case of TdP resulting from the usage of terfenadine in a female patient with liver cirrhosis and hepatocellular carcinoma (Kamisako et al., 1995). Consequently, individuals taking drugs that inhibit CYP450 enzymes face an elevated risk of cardiovascular complications when concurrently administered terfenadine (Hondeghem et al., 2011). Both LPV and ritonavir are extensively and rapidly metabolized during their first pass through the liver by CYP 3A4 isoenzyme (Cvetkovic and Goa, 2003; Yeh et al., 2006). When co-administered with LPV/r, there is a significant increase in the plasma concentration of terfenadine, leading to severe and potentially fatal events.

Previous studies showed that and LQTS may manifest during the long-term treatment of those with HIV or chronic conditions. HIV itself has been associated with a higher odds ratio of prolonged QTc (Ogunmola et al., 2015; Knudsen et al., 2019; Myerson et al., 2019). Patients with HIV may face an elevated risk of sudden cardiac death attributed to QTc interval-related cardiac events and even fatal arrhythmia, which was associated with higher levels of systemic inflammatory factors (Njoku et al., 2016; Wu et al., 2019). Furthermore, several ARV drugs, such as efavirenz (Castillo et al., 2002) and atazanavir (Chinello et al., 2007; Ly and Ruiz, 2007), have labels warning of potential QTc prolongation and TdP. In a vitro study, Anson et al. found that PIs blocked native I_Kr_ channels (Anson et al., 2005). However, other studies showed different PIs exerted minimal, if any, influence on QT interval (Charbit et al., 2009; Soliman et al., 2011). Given the prevalent inhibition of CYP450 isoenzymes by PIs and their pharmacological boosters, there is an increased risk of severe arrhythmias due to polypharmacy that may have potential cardiotoxicity in HIV patients on a PIs regimen (Back and Marzolini, 2020). Lüthi et al. (2007) described a case where LPV/r elevated the concentration of methadone, resulting in TdP. Combining antifungal drugs, like voriconazole, with LPV/r could lead to bidirectional interactions (Mikus et al., 2006), increasing the risks of QT prolongation and/or TdP (Poluzzi et al., 2010). Hence, drugs with a narrow therapeutic window are contraindicated in patients taking PIs, especially certain fluoroquinolones and antifungals, which are commonly prescribed to HIV patients with opportunistic infections (Li et al., 2017). Besides, factors like electrolyte abnormalities (hypokalemia, hypomagnesemia, and hypocalcemia), hypothyroidism, hypothermia, and severe bradycardia can elevate the risk of drug-induced TdP or even independently cause TdP (Drew et al., 2010). Female sex is also a known risk factor for drug-induced LQTS as studies have shown that women are more susceptible to TdP than men (Gowda et al., 2004).

This patient who suffered from HIV infection previously exhibited a normal ECG and QT interval with no personal or familial history of LQT or TdP. She began to develop syncopal and arrhythmia only after initiating terfenadine above the recommended dose of 180 mg/d in conjunction with an ARV regimen including LPV/r. Although there was no report about the drug interactions between LPV/r and terfenadine, the LPV/r package inserts explicitly concurrent administration with terfenadine due to the increased risk of serious arrhythmias. This interaction can be checked on the University of Liverpool HIV Drug Interactions website (https://www.hiv-druginteractions.org/). Based on the Drug Interaction Probability Scale, it is probable that the drug interaction between LPV/r and terfenadine altered the QT interval, culminating in TdP (Horn et al., 2007). In addition, an overdose of terfenadine may have exacerbated the adverse outcomes to some extent.

With improved clinical outcomes leading to increased life expectancies, patients with HIV are expected to rely on ART for an extended period (D'Ascenzo et al., 2014). The extended lifespan also brings a heightened incidence of non-AIDS disease and various complex situations of drug interactions due to concurrent conditions (Palella et al., 2006). Therefore, how to prevent drug interactions and side effects of ARVs? Primarily, combination ART selection is pivotal to the whole therapy procedure. The ideal regimens should ensure high viral suppression rates, minimal toxicity, low pill burden, and limited drug interactions. The WHO and the European AIDS Clinical Society recommend initial regimens comprising three drugs: two nucleoside reverse transcriptase inhibitors coupled with an integrase strand transfer inhibitor or a two-drug regimen of dolutegravir/lamivudine (World Health Organization, 2021; Ryom et al., 2022). Furthermore, treatment individualization is crucial for HIV-positive patients. For those on long-term ARVs or facing complex scenarios, therapeutic drug monitoring can enhance therapeutic efficacy while reducing adverse effects. This process ensures plasma drug concentrations remain within a therapeutic window via individualization of drug dosage (Carbonara et al., 2009). Drug-drug interactions are also individualized and with genetic polymorphisms (Calcagno et al., 2017). Some researchers have observed PXR gene mutation could influence CYP3A4 and CYP2B6 promoter activity, potentially exacerbating the unpredictability of drug interactions (Svärd et al., 2010). It is essential to check for drug interactions. All HIV patients should consult their HIV specialist before initiating new medication. Lastly, healthcare providers must recognize prolonged QTc interval is a potential indicator of increased cardiovascular risk. They should exercise caution when prescribing potentially QT-prolonging medications to HIV patients. In addition to terfenadine, various drugs with potential cardiotoxicity are often prescribed in the emergency department and inpatient setting. Before medication adjustment or selection, prescribers should carefully consider and rule out potential risk factors for cardiotoxicity, such as inherited long QT syndrome, advanced age, cardiovascular conditions, and certain electrolyte imbalances. Given the complex condition of HIV patients, clinical pharmacists would play an important role in evaluating the risk of ARV utility and aiding in the avoidance of irrational drug-drug interactions.

## 4 Conclusion

The real threat of arrhythmia among HIV-positive patients is frequently overlooked. With the increasing complexity of co-medication in these individuals, complications arising from drug interactions, especially those related to ARV medications, pose a significant yet underestimated threat. Numerous drugs carry the risk of inducing QT prolongation and/or TdP, whether as isolated agents or in combination with other medications. Patients on LPV/r and other PIs should be cautious of drugs with a narrow therapeutic window, especially those interacting with the CYP 3A4 enzyme system. For individuals already on medications known to induce TdP, particularly if they have other associated risk factors, it is essential to closely monitor the QTc interval, ensuring it remains normal (<500 ms). Periodic ECG assessments to identify potential irregularities are equally crucial.

## Data Availability

The original contributions presented in the study are included in the article/Supplementary Material, further inquiries can be directed to the corresponding authors.
